# Significant Reduction in *Helicobacter pylori* Load in Humans with Non-viable *Lactobacillus reuteri* DSM17648: A Pilot Study

**DOI:** 10.1007/s12602-014-9181-3

**Published:** 2014-12-07

**Authors:** Caterina Holz, Andreas Busjahn, Heidrun Mehling, Stefanie Arya, Mewes Boettner, Hajar Habibi, Christine Lang

**Affiliations:** 1 ORGANOBALANCE GmbH, Gustav-Meyer-Allee 25, 13355 Berlin, Germany; 2 HealthTwiST GmbH, Lindenberger Weg 80, 13125 Berlin, Germany; 3 Experimental and Clinical Research Center, Charité Campus Berlin-Buch (CCB), Lindenberger Weg 80, 13125 Berlin, Germany

**Keywords:** *Helicobacter pylori*, *Lactobacillus reuteri*, Selective binding, Co-aggregation, Urea breath test, Antibiotic-free therapy

## Abstract

Reducing the amount of *Helicobacter pylori* in the stomach by selective bacterial–bacterial cell interaction was sought as an effective and novel method for combating the stomach pathogen. *Lactobacillus reuteri* DSM17648 was identified as a highly specific binding antagonist to *H. pylori* among more than 700 wild-type strains of *Lactobacillus* species. Applying a stringent screening procedure, the strain DSM17648 was identified as selective binder to *H. pylori* cells under in vivo gastric conditions. The strain DSM17648 co-aggregates the pathogen in vivo and in vitro. The specific co-aggregation occurs between *Lact. reuteri* DSM17648 and different *H. pylori* strains and serotypes, as well as *H. heilmannii*, but not with *Campylobacter jejuni* or other commensal oral and intestinal bacteria. *Lact. reuteri* DSM17648 was shown in a proof-of-concept single-blinded, randomized, placebo-controlled pilot study to significantly reduce the load of *H. pylori* in healthy yet infected adults. Reducing the amount of *H. pylori* in the stomach by selective bacterial–bacterial cell interaction might be an effective and novel method for combating the stomach pathogen. *Lact. reuteri* DSM17648 might prove useful as an adhesion blocker in antibiotic-free *H. pylori* therapies.

## Introduction

*Helicobacter pylori* is a recognized pathogen and carcinogen causing gastritis, ulcers and gastric cancer. More than 50 % of the world population is infected with this stomach bacterium [1, 2]. Severity of the clinical manifestations of the infection is associated with bacterial load [3–5]. Currently, the only therapeutic option is eradication of the pathogen by a combination of several antibiotics and a proton-pump inhibitor (triple therapy; Maastricht IV/Florence Consensus Report) [6]. Eradication therapy is associated with severe side effects and development of antibiotic resistances [7]. Reducing the amount of *H. pylori* in the stomach by selective bacterial–bacterial surface interaction represents an alternative method for combating the stomach pathogen. Specific co-aggregation has been widely discussed as a means to return to homeostasis in diseased states [8–10]. While *H. pylori* resides in the mucus where it is present in its motile form, mucus is constantly produced by the epithelium and shed into the stomach lumen. This continuously releases planktonic *H. pylori* cells into the stomach. The *Lactobacillus* strain identified in this study specifically captures such *H. pylori* cells. As spray-drying or freeze-drying procedures allow the retention of binding activity, structures in the cell wall unaffected by the drying procedures are supposed to be responsible for the aggregation activity.

Previous papers describing the use of *Lact. reuteri* in *H. pylori*-related clinical studies show a reduction in *H. pylori*-associated urease activity by *Lact. reuteri* strain ATCC55730 [11]. Dore et al. [12] showed that the oral application of *Lact. reuteri* strain DSM17938, when used in combination with pantoprazole twice a day for 8 weeks, resulted in significant reduction in the urease breath test. Emara et al. [13] used a *Lact. reuteri* preparation (a mixture of strains *Lact. reuteri* DSM17938 and *Lact. reuteri* ATCC PTA6475 in combination with a triple therapy). The *Lactobacillus* supplementation increased the Gastrointestinal Symptom Rating Scale (GSRS) score significantly, but did not improve the eradication rate.

The aim of the present study was to characterize the binding activity of *Lact. reuteri* strain DSM17648 to *H. pylori* in vitro and to determine the impact of 14 days of oral intake of lyophilized *Lact. reuteri* DSM17648 cells (non-viable) on *H. pylori* load in a single-blinded, placebo-controlled study.

## Materials and Methods

### Microorganisms and Cultivation

*Lactobacillus* strains (strains from ORGANOBALANCE GmbH, Berlin, Germany) were grown in Lactobacillus MRS medium [14] at 37 °C, and *H. pylori* DSM21031 and *Campylobacter jejuni* DSM 4688T were grown in Brucella/FBS broth (BD, Heidelberg, Germany, with 10 % (v/v) fetal bovine serum, Biochrom, Berlin, Germany) at 37 °C in microaerobic atmosphere. Other *Helicobacter* species were grown in Brucella/FBS broth containing additionally 0.75 % (v/v) Vitox supplement (Oxoid, Wesel, Germany) [15].

The taxonomic identification of the *Lactobacillus* strains to the species level relied on 16 S-rDNA sequence analysis (sequencing done LCG Genomics, taxonomic classification done by Nadicom, Karlsruhe, Germany) using the primers 27f (5′-AGAGTTTGATCMTGGCTCAG-3′) and 1492r (5′-ACGGYTACCTTGTTACGACTT-3′) [16] and on phenotypic characterization using the API 50 CH system and apiweb™ software (bioMerieux, France). Bacterial counts were determined from calibration curves of optical density versus microscopic cell counts using a Neubauer chamber (Carl Roth, Karlsruhe, Germany).

### Chemicals and Enzymes

Sugars, sugar substitutes and inorganic chemicals were reagent grade (Merck, Darmstadt; Carl Roth, Karlsruhe; Germany), and proteases (protease from *Streptomyces griseus*, proteinase K from *Tritirachium album*, trypsin from bovine pancreas and pepsin from bovine pancreas) were of the highest commercially available grade (Sigma, Taufkirchen, Germany).

### Screening for Co-aggregates

**Fig. 1:**
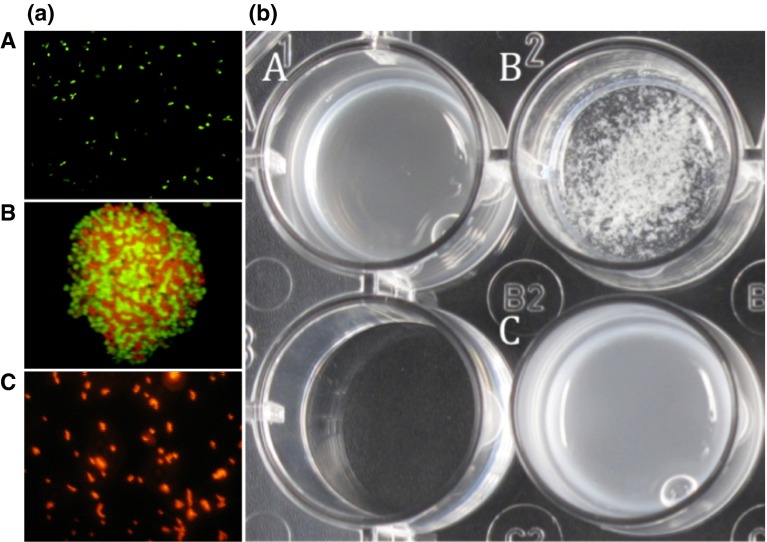
**a** Microscopic analysis of co-aggregation of *Lact. reuteri* DSM17648 with *H. pylori* DSM21031 in artificial stomach juice (pH 4), **A** *H. pylori* DSM21031 stained with hexidium iodide. **B** *Lact. reuteri* DSM17648 stained with CFDA. **C** Co-aggregate showing clumping of both strains. Bright field fluorescence microscopy (OLYMPUS BX60 microscope, 100-W mercury lamp U-RFL-T, Olympus, Japan), magnification ×1000. **b** Co-aggregation of *Lact. reuteri* DSM17648 with *H. pylori* DSM21031 is macroscopically visible. **A** *H. pylori* DSM21031; **B** *H. pylori* DSM21031 and *Lact. reuteri* DSM17648 co-aggregates; **C** *Lact. reuteri* DSM17648

Co-aggregation was performed with stationary-phase cells of lactobacilli (A600 = 4, in PBS) and *H. pylori* (A600 = 2, in artificial gastric juice pH 4, 0.3 % (w/v) pepsin, 0.5 % (w/v) sodium chloride [17]). Cells were mixed and immediate flocculation was observed. Co-aggregates could be observed visually as flocking structures, whereas no such structures were present in controls of the single strains (see also Fig. 1). If no aggregates were detected after 10 min, pairs were judged as non-co-aggregating.

For some experiments, cells were stained separately using either hexidium iodide (HI, 10 µg/mL) or carboxyfluorescein diacetate succinimidyl ester (CFDA-SE 1 µg/mL) (Invitrogen, Carlsbad, CA, USA) according to the manufacturer’s instructions. Excess dye was removed by extensive washing with PBS. Equal amounts of cells were mixed and vortexed for 10 s prior to phase-contrast and fluorescence microscopy [10].

### Flow Cytometry

*Lactobacillus* cells (without staining) and *H. pylori* cells stained with CFDA-SE were used for co-aggregation by mixing suspensions of the strain DSM17648 and *H. pylori* strain DSM21031^T^ in a ratio of 1:1 (cell/cell) to a final volume of 100 µL and subsequent shaking for 15 min. The mixture was added to 990 µL 0.5 % (v/v) sodium chloride (pH 4) in FACS tubes (BD, Heidelberg, Germany). A non-co-aggregating *Lactobacillus* strain was used as a negative control. Samples were analyzed using a flow cytometer (FACSCalibur, BD, Heidelberg, Germany). Cell co-aggregation was quantified by determining events with a high fluorescence intensity (>5 × 10^2^) via channel FL1-H (Ex 488 nm, Em 530/30) [18, 19].

### Scanning Electron Microscopy (SEM)

Cells were prepared as described above and re-suspended in PBS. Co-aggregation was induced by mixing suspensions of the strain DSM17648 and *H. pylori* strain DSM21031^T^ at a ratio of 1:1 (cell/cell). After 20-min incubation at room temperature, the resulting co-aggregates were pelleted by centrifugation (7,150×*g*, 1 min, Hettich Mikro 22R, Tuttlingen, Germany). The supernatant was carefully discarded, and co-aggregates were either frozen in liquid nitrogen, freeze-dried and sputtered with palladium (1,800× picture) or fixed in 4 % glutaraldehyde, dehydrated in graded ethanol solutions, dried in liquid CO_2_ and sputtered with palladium (11,000× picture) before SEM. SEM was done using a FEI Quanta 200 FEG Field emission scanning electron microscope. Some images were colorized according to the bacillary or spiral shape to facilitate viewing (eye of science Meckes and Ottawa GbR, Reutlingen, Germany).

### Sugar and pH Effects on Co-aggregation

Co-aggregation was tested in the presence of 25 mM sucrose with known co-aggregating pairs. Analogous simultaneous incubations were done testing lactose, glucose, maltose, iso-maltose, fructose or sorbitol to detect possible interference with the ability of the strain DSM17648 to co-aggregate *H. pylori*. To evaluate the pH dependency of co-aggregation, DSM17648 cells were re-suspended in 0.1 M Sorensen buffer (0.1 M glycine, 0.1 M NaCl) adjusted to pH 2.0 and in McIlvaine citrate–phosphate buffer (0.1 M citrate, 0.2 M disodium phosphate) adjusted to pH values from 3.0 to 8.0, in 1.0 pH unit intervals. *H. pylori* cells were re-suspended in artificial gastric juice [17]. Co-aggregation was then assessed in a ratio of 1:1 (DSM17648/*H. pylori*). pH values of final mixtures were controlled. No pure cultures evidenced auto-aggregation within this pH range.

### Protease Pre-treatment

The strain DSM17648 and *H. pylori* were grown separately to stationary phase, harvested by centrifugation, washed in PBS, and 1 mL aliquots adjusted to A600 = 4 (for DSM17648) or A600 = 2 (for *H. pylori*) in monopotassium phosphate/calcium chloride buffer (pH 7.0) containing either one of four proteases: protease *Strep. griseus* Type XIV (5.7 U/mg), proteinase K (51 U/mg), trypsin (40 U/mg) or pepsin (2,950 U/mg) at a final concentration of 2.0 mg/mL. After incubation for 1 h at 37 °C, cells were washed, re-suspended again in PBS (pH 7.0), and 500 µL aliquots of each preparation mixed, and co-aggregation assayed visually and microscopically.

### Spray Drying and Lyophilization of Cells

Spray drying of cells was done using a Büchi B-191 spray dryer, inlet temperature 140 °C, aspiration 75 % and pump rate 5 % [20]. NaCl [75 % (w/w) final concentration] was used as carrier substance. Before lyophilization, cells were washed, re-suspended in 15 % (w/v) skimmed milk powder and frozen at −80 °C. Lyophilization was done under vacuum (0, 1 mbar) for 24 h [21].

### Study Population

The original setting of the study was a placebo-controlled co-twin control design with one twin receiving the active treatment while the co-twin received a placebo. Concordance rates for *H. pylori* infection in monozygotic twins have been reported at 80 % [22], whereas in dizygotic twins they are 60 %. Heritability for quantitative levels of *H. pylori* colonization has been estimated at 0.8. Historical prevalence of infection by *H. pylori* for the general population was reported as 45 %, although more recent studies suggest a reduction to approximately 25 % in the Western world [23, 24]. For Germany, a prevalence of 39 % was reported in 1996 [25]. Based on those figures, the first screening phase was planned to include analysis for 64 twin pairs, expecting 29 pairs with at least one affected twin, and 23 concordant pairs, i.e., pairs with positive findings for both twins. As incidence rates found in the screening phase were lower than expected from published figures, the original design was then adapted to include singletons in a pre–post design. A second screening phase included twins as well as singletons. Subjects were included if they had reached the age of 18 and had a positive *H. pylori* finding in the ^13^C urea breath test (Helicobacter Test INFAI^®^, Dd ≥ 4 ‰). Informed consent was obtained from all persons for being included in the study. Additional informed consent was obtained from all patients for which identifying information is included in this article. Exclusion criteria were any medication interfering with the action of the lactobacilli, previous surgical procedures affecting stomach or small intestine with potential interference with the study, e.g., gastrectomy or gastric bypass, diabetes type 1 or 2, familiar lipid metabolism diseases, any other major disease, weight changes >3 kg over the last 3 months, pregnancy or lactation, alcohol or drug abuse, or psychiatric diseases.

### Study Protocol

The study was approved by the local ethics advisory committee (Charité, Berlin, Germany) and was conducted according to the Declaration of Helsinki [26]. As the trial was not a clinical trial, the trial was not registered, as at the time of the trial in Germany it was not customary for pilot type trials to be registered.

The test product (active ingredient) consisted of lyophilized dead cells of the strain DSM17648, prepared as solid tablets for oral application. Each tablet contained 5 × 10^9^ cells (determined by counting in Neubauer chamber), and the daily dosage of four tablets translates into 2 × 10^10^ cells. Verum and placebo tablets were identical in weight (250 mg), size, color and flavor. Within concordant affected twin pairs, treatment was randomized in parallel for a time period of 14 days. In singletons, active treatment and placebo were given in a single-blinded non-randomized crossover design. The first period of 14 days was the placebo phase; after a second breath test, active treatment was given for another 14 days, followed by a breath test. Four to six weeks after the treatment phase, a follow-up breath test was conducted. Subjects were instructed to take two tablets after breakfast as well as after their evening meal. During the treatment phase, no lifestyle or dietary changes were to be initiated and no probiotic food products or cranberries were to be used. Subjects were asked to fill in a study-specific questionnaire to document well-being, any potential side effects, smoking, alcohol use, nutrition and medication.

### Measurements

Detection of *H. pylori* infection in the screening phase and quantification of colonization to verify effects of the strain DSM17648 were accomplished by a breath test, as this diagnostic approach is best suited to screening as well as detection of intra-individual changes [27]. *Helicobacter* Test INFAI^®^ is a breath test for direct noninvasive quantitative detection of the bacterium *H. pylori* [28]. The test is based on urease activity of *H. pylori*. Specificity (98.5 %) and sensitivity (97.9 %) of *Helicobacter* Test INFAI^®^ are comparable to traditional invasive diagnostic methods (endoscopy or biopsy). As the breath test reflects the current status of colonization by *H. pylori*, it is well suited to detect reduction in or eradication of the bacteria [29, 30].

The test is based on the hydrolysis of ^13^C urea to ammonium and ^13^C-enriched carbon dioxide, which is detectable in the breath. Patients ingest a small amount of the ^13^C urea isotope. Carbon dioxide resulting from the degradation of urea contains this isotope, detectable by mass spectrometry. As there is a small amount of naturally occurring ^13^C even in the absence of urease activity, breath samples are taken before and after the ingestion of ^13^C urea. If there is no difference, the test is negative, indicating no infection with *H. pylori*. There is a quantitative relation between urease activity and amount of ^13^C in breath that indirectly relates to the level of colonization by *H. pylori*.

### Statistics

All historical and clinical data were entered into a dedicated trial database. Statistical analysis was conducted using SPSS version 16.0.2. We computed differences in ^13^C urea breath test (UBT) values against initial measurements: ΔActive = ^13^C UBT Active − ^13^C UBT Initial, ΔPlacebo = ^13^C UBT Placebo − ^13^C UBT Initial, ΔWash-out = ^13^C UBT Wash-out − ^13^C UBT Initial. Additionally, the absolute test values between the various study time-points were compared: ^13^C UBT Initial, ^13^C UBT Verum (after 14-day verum treatment), ^13^C UBT Placebo (after 14-day placebo treatment), ^13^C UBT Wash-out (4–6 weeks after verum treatment). Data from twin pairs were combined and analyzed as in singletons. The co-twin control design is comparable to a crossover design, but without any potential carry over effects. There was no randomized order for verum/placebo treatment, as no continuing placebo effect was expected.

All data were tested for deviations from normal distribution by Kolmogorov–Smirnov test. Mean differences were computed by pairwise *t* test. Potential relations between response to treatment and initial level of colonization were explored by linear regression. An error level of 5 % was set as threshold for significance. Results are reported as mean ± standard deviation (SD); figures present the standard error of the mean (SEM).

## Results

### Co-aggregation Analysis of *Lact. reuteri* DSM17648

**Fig. 2:**
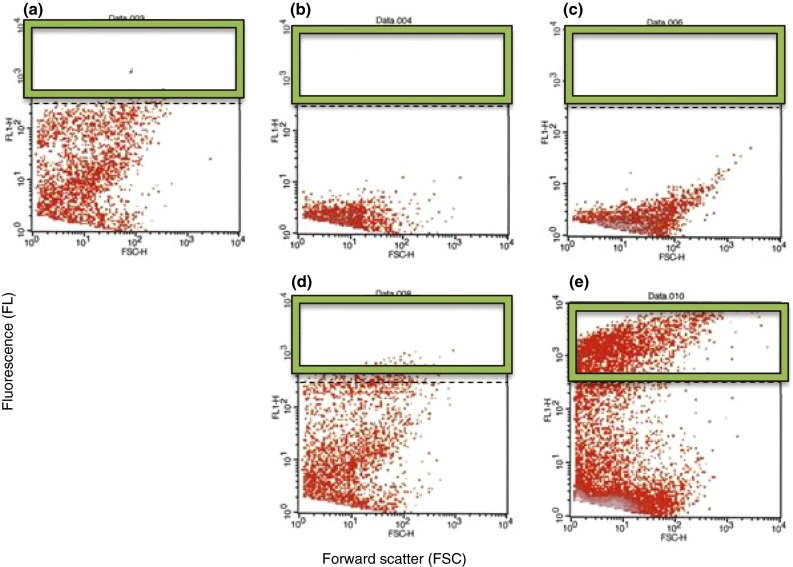
Co-aggregation of *Lact. reuteri* DSM17648 with *H. pylori* DSM21031 was analyzed by flow cytometry (**e**). *H. pylori* cells were CFDA stained. Samples were analyzed using flow cytometry, and cell co-aggregation was quantified by determining the events with a high FL (>5 × 10^2^, area within *green frame*). Co-aggregation was not observed when strains were analyzed separately (**a**–**c**) nor when a non-aggregating *Lactobacillus* strain was used as a control (**d**)

**Fig. 3:**
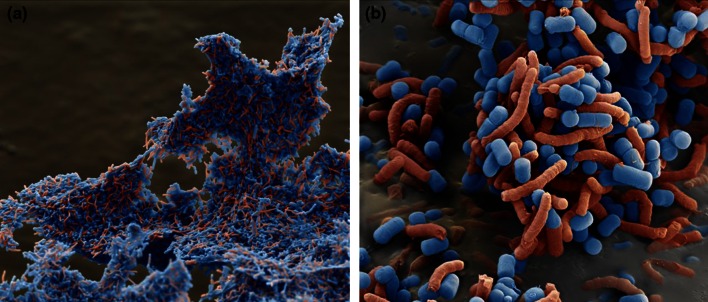
Scanning electron microscopy of co-aggregates of *Lact. reuteri* DSM17648 (*blue*) and *H. pylori* (*red*), **a** ×1,800 magnification; **b** ×11,000 magnification. Some images were colorized according to the bacillary or spiral shape to facilitate viewing

Lactobacilli that co-aggregate *H. pylori* were sought among a large *Lactobacillus* strain collection. The in-house, private strain collection has been assembled from wild-type strains of diverse origin, such as food sources, plants, vegetables or human skin. Strains are classified according to the physiological characteristics prior to being included in the screening process. Among 700 *Lactobacillus* strains tested, only eight were found to co-aggregate with spiral forms of *H. pylori* strain DSM21031, without exhibiting any auto-aggregation (Fig. 1). Three of the co-aggregating lactobacilli (strains DSM17648, DSM17647 and DSM17651) were identified as *Lact. fermentum* (API method). One of these—*Lact. fermentum* DSM17648 [classified as *Lact. reuteri* by 16 S-rDNA sequencing and sequence alignment (100 % identical to accession numbers CP000705, CP006603, CP006011 (at 99 % coverage))]—was analyzed in depth (Table 1). Numerous other *Lact. fermentum* and *Lact. reuteri* strains were tested in parallel for auto-aggregation and co-aggregation under the described conditions. None of them formed co-aggregates with *H. pylori*. *Lactobacillus* and *Helicobacter* strains did not auto-aggregate (Fig. 1). To confirm that both species were present in the aggregates, cells were stained separately using either hexidium iodide or carboxyfluorescein diacetate succinimidyl ester. Both the strain DSM17648 and *H. pylori* DSM21031 participated in the aggregation (Fig. 1). Co-aggregation occurs within seconds after mixing the strains. Quantification of co-aggregate formation between *Lact. reuteri* DSM17648 and *H. pylori* DSM21031 by flow cytometry (Fig. 2) shows that one *Lactobacillus* cell binds 2–3 *Helicobacter* cells. Interestingly, the co-aggregation activity is preserved during lyophilization or spray drying of whole cells of *Lact. reuteri* DSM17648 and persists in non-viable cells (Table 3). Spray-dried or lyophilized cells of strain DSM17648 induced co-aggregate formation with the same sensitivity as untreated cells. Expression of co-aggregation activity is dependent on the growth phase of *Lact. reuteri* DSM17648, and it is present at entry into stationary growth and during stationary phase. SEM images of co-aggregates were prepared to analyze cellular sites of the attachment. Figure 3 shows that single cells of the strain DSM17648 bind several *H. pylori* cells resulting in cross-linking of the co-aggregates. Binding sites on the cells of the strain DSM17648 appear evenly distributed over the cell surface, and binding sites on *H. pylori* cells do not seem to be present on flagellar structures.

**Table 1:** Aggregation of *H. pylori* by lactobacilli is *Lactobacillus* strain specific

*Lactobacillus* strain	Co-aggregation of *H. pylori* type strain DSM21031^T^	Reference
*Lact. fermentum* ^a^ */reuteri* ^b^ OB-LbHp-1 (DSM17648)	+	This work; ORGANOBALANCE strain collection
*Lact. fermentum* ^a^ */reuteri* ^b^ OB-LbHp-2	+	This work; ORGANOBALANCE strain collection
*Lact. fermentum* ^a^ */reuteri* ^b^ OB-LbHp-3	−	This work; ORGANOBALANCE strain collection
*Lact. fermentum* ^a^ */reuteri* ^b^ OB-LbHp-4	−	This work; ORGANOBALANCE strain collection
*Lact. reuteri* ^b^ DSM 17509	−	DSMZ Braunschweig, Germany
*Lact. reuteri* DSM 20053	−	DSMZ Braunschweig, Germany
*Lact. reuteri* DSM 20056	−	DSMZ Braunschweig, Germany
*Lact. fermentum* ^ab^ OB-LbHp-5	−	This work; ORGANOBALANCE strain collection
*Lact. fermentum* ^ab^ OB-LbHp-6	−	This work; ORGANOBALANCE strain collection
*Lact. fermentum* ^ab^ OB-LbHp-7	−	This work; ORGANOBALANCE strain collection
*Lact. fermentum* ^ab^ OB-LbHp-8	+	This work; ORGANOBALANCE strain collection
*Lact. fermentum* ^ab^ OB-LbHp-9	+	This work; ORGANOBALANCE strain collection

^a^Identified by API;^b^ identified by 16 S rDNA sequence

**Table 2:** *Lact. reuteri* strain DSM17648 co-aggregates different types and species of *Helicobacter*, but not *Campylobacter* and bacterial representatives of oral or intestinal flora

Strains	Co-aggregation by *Lact. reuteri* DSM 17648
*H. pylori* DSM 9691, type I	+
*H. pylori* DSM 10242, type I	+
*H. pylori* DSM 21031^T^, type I	+
*H. pylori* CCUG 19106, type II	+
*H. heilmannii* ATCC 49286, type 1	+
*H. heilmannii* DSM 24751^T^, type 2	+
*H. canis* CCUG 32756	+
*Campylobacter jejuni* DSM 4688^T^	−
*Streptococcus salivarius* DSM 20560^T^	−
*Clostridium leptum* DSM 753^T^	−
*Clostridium saccharolyticum* DSM 2544^T^	−
*Bacteroides fragilis* DSM 2151^T^	−
*Escherichia coli* DSM 30083^T^	−

^T^Type strains

**Table 3:** Co-aggregation activity is present in nonviable cells of *Lact. reuteri* DSM17648

	Culture	Lyophilized cells	Spray-dried cells^a^
Colony forming units (mL^−1^ or mg^−1^)	2.0 × 10^9^	3.0 × 10^7^	0
Co-aggregation	+++	+++	+++

^a^Cells were spray-dried and incubated at 40 °C for 24 h

As the interaction between cells of *Lact. reuteri* DSM17648 and *H. pylori* DSM21031 involves cell surfaces, we tested for possible interference by surface modulating treatments. Co-aggregation persists in the presence of sugar (sucrose, lactose, glucose, fructose, maltose, isomaltose, and sorbitol). It occurs at comparable efficiency at room temperature and at 37 °C, and co-aggregation activity is observed over a wide pH range (pH 2.0—corresponding to empty stomach conditions—up to pH 8, including typical pH values after meals). No pure cultures evidenced auto-aggregation within this pH range. Slightly smaller co-aggregates formed at pH 2 compared with pH 8 in vitro. Thus, aggregation of *H. pylori* by *Lact. reuteri* DSM17648 occurs at pH values and conditions encountered in the human stomach.

The susceptibility to protease inactivation of the co-aggregation determinants on the surfaces of both the strain DSM17648 and *H. pylori* DSM21031 was tested after treatment with protease *Strep. griseus* Type XIV, proteinase K, trypsin or pepsin. Incubating *Lact. reuteri* DSM17648 with any protease before co-aggregation reduced binding to *H. pylori* DSM21031 by 30 %, but did not eliminate it completely. *H. pylori* required pretreatment with the protease pepsin (as is naturally present in gastric fluids) to be fully active in co-aggregation with the strain DSM17648.

*Lactobacillus reuteri* DSM17648 does not co-aggregate with common non-*Helicobacter* members of the human flora. Neither the major intestinal commensals nor *C. jejuni* detectably co-aggregate with *Lact. reuteri* DSM17648 (Table 2), and no auto-aggregation was observed. Co-aggregation is active with different *H. pylori* strains (type I and type II strains) as well as with *H. heilmannii* strains (type I and type II) and with *H. canis* of animal source. Thus, the strain DSM17648 specifically co-aggregates *H. pylori* without interfering with other bacteria of the commensal intestinal flora.

### Pilot Study

**Fig. 4:**
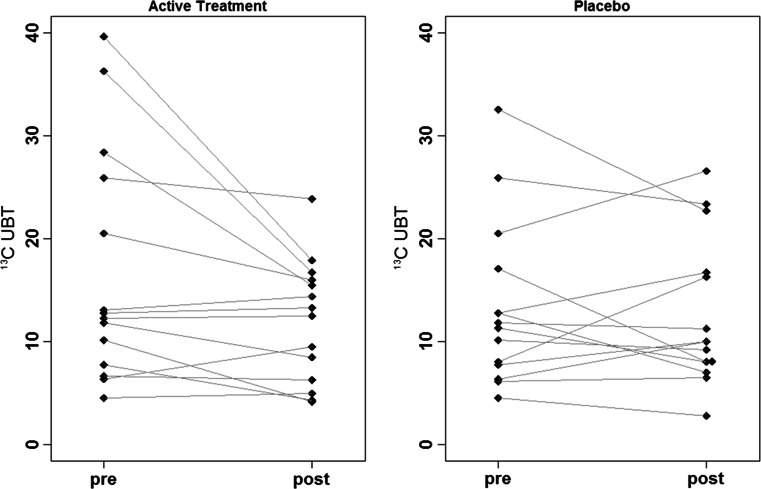
Absolute ^13^C UBT values of individuals before and after treatment with verum and placebo

The strain DSM17648 was used in a placebo-controlled pilot study to evaluate the effect of the strain DSM17648 in asymptomatic *Helicobacter*-positive test persons after a two-week application. Screening included 128 subjects, 47 twin pairs and 34 singletons; 27 subjects had a positive breath test result. Overall *Helicobacter* prevalence was 21 %; 6 twin pairs were concordant and 10 pairs discordant positive. Fourteen independent treatments were started with no dropouts during the trial phase. All 6 concordant twin pairs participated in the study, as well as 4 discordant twin pairs and 4 singletons. Due to the large inter-individual variability of quantitative measures of colonization (^13^C UBT Initial), analysis of *H. pylori* reduction by the strain DSM17648 was primarily based on intra-individual changes after active treatment or placebo (Δverum vs. Δplacebo). Treatment by placebo did not result in a significant change in ^13^C UBT (Δplacebo −0.6 ± 5.3), whereas verum treatment significantly reduced ^13^C UBT values (Δverum −4.9 ± 7.8, *p* = 0.026 vs. placebo), indicating significant reduction in *H. pylori*. Absolute values of ^13^C UBT at baseline measurement, after placebo and after verum treatment were 14.1 ± 9.9, 12.7 ± 7.2 (ns vs. initial) and 11.9 ± 5.9 (*p* vs. initial 0.01, *p* vs. placebo 0.03), respectively. To allow for a detailed evaluation of response to the strain DSM17648, individual values for ^13^C UBT are plotted in Fig. 4. After verum treatment, the majority of subjects showed a reduction in *H. pylori* colonization. Responses showed some variability, from no reduction to a delta of more than 20. In comparison, after 2 weeks of placebo, some subjects had lowered values while others had increases in the same magnitude, indicating no systematic effect.

**Fig. 5:**
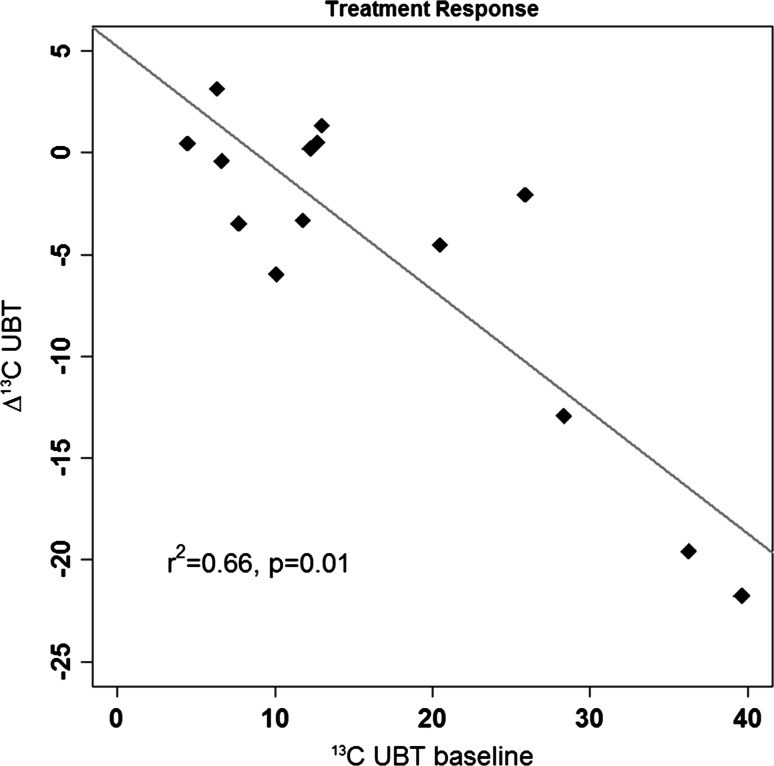
Linear regression between initial values and response to treatment (verum). Response is significantly stronger with increased basal *H. pylori* colonization level

Values of ^13^C UBT after wash-out (*x* ± *y*) are not significantly different from verum treatment values. The effect of reduced *Helicobacter* values lasts over the actual treatment. There was some dependency of treatment response on initial values (*r* ^2^ = 0.66, *p* = 0.01, Fig. 5). With increasing level of colonization, the lowering effect caused by the strain DSM17648 becomes stronger. For placebo treatment, the same dependency was found albeit to a lesser degree (*r* ^2^ = 0.35, *p* = 0.02), probably reflecting regression to the mean effects. A direct placebo effect on immune response cannot be ruled out. This potential effect is significantly lower than the specific action of the strain DSM17648. During the course of the study, there was neither any change in life style, e.g., in physical activities or diet, nor health as indicated by the questionnaire. No side effects were reported in either study group.

## Discussion

The specific and fast co-aggregation of defined *Lactobacillus* strains of the species *Lact. reuteri/fermentum*, *Lact. brevis* and *Lact. pentosus* (typified *Lact. reuteri* DSM17648) with *H. pylori* under gastric conditions is novel. Co-aggregation of *Lact. reuteri* DSM17648 also occurs with *H. heilmannii* and *H. canis*. Major commensals in the intestinal environment are not co-aggregated by *Lact. reuteri* DSM17648. Numerous other lactobacilli in a very large collection of *Lactobacillus* wild-type strains (collected from various natural habitats) including many other *Lact. fermentum*, *Lact. brevis* and *Lact. pentosus* strains fail to manifest *H. pylori*-specific co-aggregation. Co-aggregation activity is not affected by sugars and pH (over a wide range between 2 and 8), and it requires a pepsin pretreatment of spiral form cells of *H. pylori*. It does not occur with far less infective coccoidal *H. pylori* cells [31], and it is dependent on a *Lact. reuteri* DSM17648 cell surface factor that is present at the end of the exponential growth phase and during stationary phase. Co-aggregation between *H. pylori* and the strain DSM17648 occurs within seconds. While Chen et al. [32] observed a very slow interaction between some lactobacilli from food source, the present paper is to our knowledge the first description of a rapid and efficient co-aggregation of *H. pylori* by a specific *Lactobacillus* strain under gastric conditions. It is proposed that *Lact. reuteri* DSM17648 interferes with mobility of *H. pylori* and its adherence to the gastric mucosa by entangling the cells into aggregates and masking *H. pylori* surface sites that are ordinarily available for binding to human epithelium. Once bound, co-aggregates will be flushed from the stomach by natural bowel movement. Interestingly, the aggregation activity was preserved when the cells were killed by freeze drying or spray drying. It can be assumed that binding is due to specific surface molecules on the *Lact. reuteri* DSM17648 cells which are strain specific and are resistant to such process steps. Such surface molecules might include lipoteichoic acid and carbohydrate structures.

This novel anti-*H. pylori* activity has not been described previously as a mode of action for probiotic treatment of *H. pylori* infections. This hypothesis was tested in a proof-of-concept in vivo study. Our data demonstrate the significant decrease in *H. pylori* load by a two-week application of *Lact.* *reuteri* DSM17648 in healthy subjects with detectable *H. pylori* infection in a general population sample. The principal outcome criterion was the reduction in *H. pylori* as measured by ^13^C urease breath test (Helicobacter Test INFAI^®^) after a 14-day supplementation period of *Lact. reuteri* DSM17648 at a daily dose of 2 × 10^10^ non-viable lyophilized cells. Data obtained in a parallel clinical study support the data reported in this paper [33]. Previous studies with non-specific probiotics require the application of live microorganisms while *Lact. reuteri* DSM17648 is active as non-viable cell preparation. This will greatly reduce any potential side effects and will help ensure stable activity in a potential consumer product and in pharmaceutical and medicinal formulations.

Our study reveals a novel *Lact. reuteri* strain (the strain DSM17648) that features unique properties as it specifically aggregates with planktonic *H. pylori* in the stomach. Freeze-dried (and spray-dried) preparations significantly reduce the *H. pylori* load (measured by urease breath test) after a 14-day oral treatment period in *H. pylori*-positive test persons.

*Lact. reuteri* strain DSM17648 can become a central part of a strategy to avoid using antibiotics and combating antibiotic resistances in *H. pylori* infections, in reducing *H. pylori* load, either as a prophylactic food additive or a medical cure to treat *H. pylori*-induced stomach diseases.
